# Neuroimaging Correlates of Suicidality in Decision-Making Circuits in Posttraumatic Stress Disorder

**DOI:** 10.3389/fpsyt.2019.00044

**Published:** 2019-02-12

**Authors:** Jennifer Barredo, Emily Aiken, Mascha van 't Wout-Frank, Benjamin D. Greenberg, Linda L. Carpenter, Noah S. Philip

**Affiliations:** ^1^Center for Neurorestoration and Neurotechnology, Providence VA Medical Center, Providence, RI, United States; ^2^Department of Psychiatry and Human Behavior, Alpert Medical School of Brown University, Providence, RI, United States; ^3^Neuromodulation Research Facility, Butler Hospital Providence, Providence, RI, United States

**Keywords:** suicide, neuroimaging, functional connectivity, morphometry, decision-making, cognitive control, suicidality, posttraumatic stress disorder

## Abstract

In depression, brain and behavioral correlates of decision-making differ between individuals with and without suicidal thoughts and behaviors. Though promising, it remains unknown if these potential biomarkers of suicidality will generalize to other high-risk clinical populations. To preliminarily assess whether brain structure or function tracked suicidality in individuals with posttraumatic stress disorder (PTSD), we measured resting-state functional connectivity and cortical thickness in two functional networks involved in decision-making, a ventral fronto-striatal reward network and a lateral frontal cognitive control network. Neuroimaging data and self-reported suicidality ratings, and suicide-related hospitalization data were obtained from 50 outpatients with PTSD and also from 15 healthy controls, and all were subjected to seed-based resting-state functional connectivity and cortical thickness analyses using *a priori* seeds from reward and cognitive control networks. First, general linear models (GLM) were used to evaluate whether ROI-to-ROI functional connectivity was predictive of self-reported suicidality after false discovery rate (FDR)-correction for multiple comparisons and covariance of age and depression symptoms. Next, regional cortical thickness statistics were included as predictors of ROI-to-ROI functional connectivity in follow-up GLMs evaluating structure-function relationships. Functional connectivity between reward regions was positively correlated with suicidality (*p*-FDR ≤ 0.05). Functional connectivity of the lateral pars orbitalis to anterior cingulate/paracingulate control regions also tracked suicidality (*p*-FDR ≤ 0.05). Furthermore, cortical thickness in anterior cingulate/paracingulate was associated with functional correlates of suicidality in the control network (*p*-FDR < 0.05). These results provide a preliminary demonstration that biomarkers of suicidality in decision-making networks observed in depression may generalize to PTSD and highlight the promise of these circuits as transdiagnostic biomarkers of suicidality.

## Introduction

We are in the midst of a public health crisis. Suicide rates have risen precipitously over the last decade in most demographic groups (1). Despite investments in research, the ability to predict patients' suicide risk remains poor (2, 3). Problems with risk assessment are due, in part, to dependence upon patients' insight and willingness to disclose suicidal thoughts and behaviors. Thus, there is an immediate need to identify novel, objective biomarkers of risk.

Interest in the link between suicidality and decision-making emerged from the frequent observation of impulsive or “short-sighted” behavior in psychiatric populations at high risk for suicide [see (4)] and the prevalence of suicidality in behaviors associated with excessive risk-taking [e.g., gambling (5) and substance use (6)]. Several studies in patients with depression have found that those with a history of prior suicide attempts and depression make more high-risk choices on value-based decision-tasks [e.g., simulated gambling (7, 8), delay discounting (9, 10), probabilistic learning (11)] when compared to depressed, non-suicidal counterparts [but see (12)].

**Figure 1:**
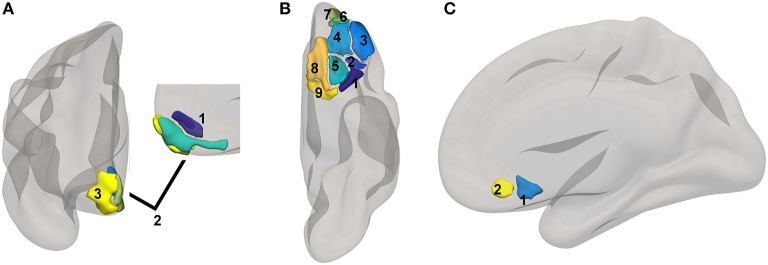
Reward network ROI locations. ROIs based upon a subset of regions involved in reward from the high-dimensional atlas Human Connectome Project Multimodal Atlas (13). Surface-space ROIs were converted into volumetric Montreal Neurologic Institute Atlas space with FreeSurfer (14). **(A)** medial PFC regions: 1. 10r, 2. 10v, 3. 10pp. **(B)** orbital regions: 1. 47s, 2. 47m, 3. a47r, 4. 11l, 5. 13l, 6. a10p, 7. p10p, 8. OFC, 9. pOFC. **(C)** subgenual areas: 25, s32.

Complementary functional magnetic resonance imaging (fMRI) results from studies of depressed, previous suicide attempters consistently report that orbitofrontal cortex (OFC) activation tracks high-risk choice behavior (8, 12). OFC is part of a ventral prefrontal cortex (PFC)-to-basal ganglia reward circuit that supports adaptive decision-making by integrating reward and critical context information (Figure 1) (15). Transdiagnostic meta-analytic data indicates that OFC gray matter is lower in prior suicide attempters when compared those without suicidality (16). Moreover, both structural (10) and functional (11) correlates of sub-optimal decision-making in this reward circuit track suicidality in individuals with depression. In depression, metabolic hyperactivity in the reward network has also been shown to distinguish patients with history of suicide attempt from non-attempters (17). Collectively, these findings recommend this circuit as a source of potential transdiagnostic biomarkers of suicidality.

Neuroimaging has also identified potential biomarkers of suicidality in other decision-making circuits, namely those that sub-serve general cognitive control. Cognitive control processes bias neural operations underlying thought and action toward a desired goal or outcome (18). Cognitive control is particularly important when obtaining or maximizing long-term benefits involves overriding the drive toward immediate reinforcement. The lateral PFC and anterior cingulate cortex (ACC) regions are strongly associated with cognitive control (18) (Figure 1). Importantly, the cortico-basal ganglia-thalamic loops provide the anatomical means for cognitive control regions to functionally interact with the reward network during decision-making (19, 20) (see Figure 1). Volumetric neuroimaging indicates that gray matter volume is lower in the striatum (10, 21, 22), OFC (23), and PFC control regions (21–24) in mood disordered individuals with history of suicidality, a characteristic that may impact functional network interactions and the capacity for adaptive decision-making. Indeed, fMRI signals in dorsolateral PFC differ between depressed patients with and without suicide attempt history during decision-making in gambling (12), delayed discounting (25), and reward-guided probabilistic learning tasks (11).

Though the evidence supporting the link between suicidality and decision-making is compelling, the majority of previous studies have been conducted in individuals with major depressive disorder (16, 26). It is unknown if findings will generalize to other high-risk groups. To address this gap, we examined resting-state functional connectivity in decision-making networks in a naturalistic sample of adult outpatients selected for a current diagnosis of posttraumatic stress disorder (PTSD). To further probe underlying structure-function relationships, we also tested cortical thickness in regions implicated in suicidality by our functional connectivity analysis. We hypothesized that functional connectivity within decision-making networks would positively correlate with self-reported suicidality, and that low cortical thickness may partially explain differences in functional connectivity. Previewing our results, we found that functional connectivity in the reward and cognitive control networks was positively correlated with self-reported suicidality after covariance for age and depression symptoms. We also found a significant relationship between cortical thickness in the anterior paracingulate and functional connectivity in the control network.

## Materials and Methods

### Participants

Data from 65 individuals were included. Fifty (Veterans = 36; Non-Veterans = 14) were enrolled in studies of non-invasive neuromodulation treatments for PTSD; the remainder were healthy U.S. military Veteran controls (*n* = 15). Data analyzed here were collected prior to administration of neuromodulation treatment. Participants were provided with complete details of all experimental procedures prior to study enrollment and were administered written informed consent. Study recruitment and enrollment procedures were conducted at the Providence VA Medical Center or Butler Hospital, Providence, Rhode Island. All procedures were approved by the relevant Institutional Review Board and abide by the Code of Ethics of the World Medical Association (Declaration of Helsinki) for experiments involving humans. Study exclusion criteria included MRI contraindications, bipolar I disorder, psychotic disorders, active substance abuse, or an unstable medical or neurological condition. Participants were either medication-free or taking stable doses of all medications for a minimum of 4 weeks prior to study enrollment. See Table S1 in the Data Supplement for additional medication information.

### Instruments and Assessments

Diagnostic information for psychiatric conditions was obtained using the Structured Clinical Interview for DSM-IV-TR or DSM-V (27). PTSD and depressive symptom severity were measured using the Posttraumatic Stress Disorder Checklist [PCL-5 (28)] and Inventory of Depressive Symptoms Self-Report [IDS-SR (29)], respectively. Participants' responses to IDS-SR item #18: “Thoughts of Death or Suicide” measured suicidality over the preceding 7 days. Possible responses were: (0) no thoughts of death or suicide, (1) feeling that life is empty or wondering if it's worth living, (2) thinking of suicide or death several times a week for several minutes, or (3) thinking of suicide or death in detail multiple times each day, having a specific suicide plan, or having made an attempt. To index suicidality across the lifespan, we collected information about prior psychiatric hospitalizations during the intake interviews and verified that hospitalizations were related to suicidality by chart review. See Table 1 for a breakdown of PTSD and depression symptom severity by level of suicidality, as well as additional hospitalization information.

**Table 1:** Demographics by self-reported current suicidality^a^.

	**PTSD,** **Item > 1**	**PTSD,** **Item = 1**	**PTSD,** **Item = 0**	**No PTSD,** **Item = 0**
Sample *n*	11	19	20	15
Age	51.5 (8.2)	51.4 (12.6)	45.3 (11.3)	54.7 (12.4)
Sex	f(6)	f(4)	f(7)	f(4)
Race				
White	100%	95%	83%	88%
Black	-	-	-	6%
Multiracial	-	5%	5%	6%
Education				
Some HS	-	-	12%	-
HS	46%	53%	76%	47%
Bachelor's	54%	29%	12%	20%
Post-graduate	-	6%	6%	33%
Total IDS-SR	52.2 (15.6)^*^,^**^	47.1 (11.7)^*^,^**^	35.8 (9.0)	8.5 (5.8)
Total PCL-5	53.3 (14.2)^*^,^**^	52.1 (12.9)^*^,^**^	41.1 (11.4)	5.3 (6.1)
Prior hospitalization	91%	74%	30%	0%
MDD	100%	100%	78%	-
SUD	50%	64%	57%	-
Other anxiety	25%	64%	50%	-

* Greater than non-suicidal controls at p < 0.01

** Greater than non-suicidal patients at p < 0.01

a *Statistics for age, total IDS-SR, and total PCL-5 are means and standard deviations. Prior hospitalization refers to the percentage of the subsample with self-reported lifetime history of at least one psychiatric hospitalization for suicidal risk verified by chart review. Sample size and sex are raw counts. Race is in percentage of sample size. Education refers to highest level of education completed. Major depression disorder (MDD) refers to percentage of sample meeting DSM diagnostic criteria for current MDD. DSM Substance Use Disorder (SUD) and other anxiety disorder were not available for all patients with PTSD and percentages are based on reduced sample sizes [Item>1 (n = 4); Item = 1 (n = 14), Item = 0 (n = 14)]. SUD and other anxiety information refer to meeting either current or past diagnostic criteria*.

### MRI Data Acquisition and Preprocessing

MRI was acquired at the Brown University MRI Research Facility using a Siemens 3T MRI scanner (Siemens, Erlangen, Germany), either the TimTrio or Prisma model, equipped with a 32-channel head coil. A structural T1-weighted image was collected from each participant to enable functional normalization and morphometry (cortical thickness) analysis (TR = 1,900 ms, TE = 2.98 ms, and FOV 256 mm^2^, 1 mm^3^). Immediately after this structural scan, a T2^*^-weighted gradient-echo echo-planar imaging (EPI) sequence sensitive to blood oxygenation level-dependent (BOLD) contrast was used to collect functional data (TR = 2,500 ms, TE = 28 ms, flip angle = 90 deg., FOV = 64 × 64, 42 slices, voxel size = 3.0 mm isotropic; 192 volumes). Participants were instructed to keep their eyes open and remain as still as possible during the acquisition of “resting state” functional data.

All data preprocessing and analyses used SPM12 (University College London; https://www.fil.ion.ucl.ac.uk/spm/) or the CONN toolbox [www.nitrc.org/projects/conn, RRID:SCR_009550; (30–32)] unless otherwise indicated. Preprocessing for functional images included slice-time correction, head motion estimation, realignment, functional segmentation, registration to Montreal Neurological Institute (MNI)-152 atlas space, and an artifact analysis in which high motion volumes or those where volume-to-volume global signal variance exceeded 3 standard deviations were flagged for confound regression in subsequent first-level models. Motion thresholds for exclusion were set at >0.5 mm translational or >0.02 radians rotational motion. Then, the anatomical CompCor (aCompCor) method was used to remove non-neuronal signals from fMRI data via the extraction of five principal components from white matter and CSF fMRI time courses. These principle components were regressed from subject-level data, along with the linear trend, six estimated motion parameters and their 1st derivatives, and high-motion or high global signal variance time points to limit the influence of these potential sources of spurious variance on our results. Residuals were also band-pass filtered (high-pass = 0.008, low-pass = 0.1) after confound regression.

### Region-of-Interest (ROI) Selection

**Figure 2:**
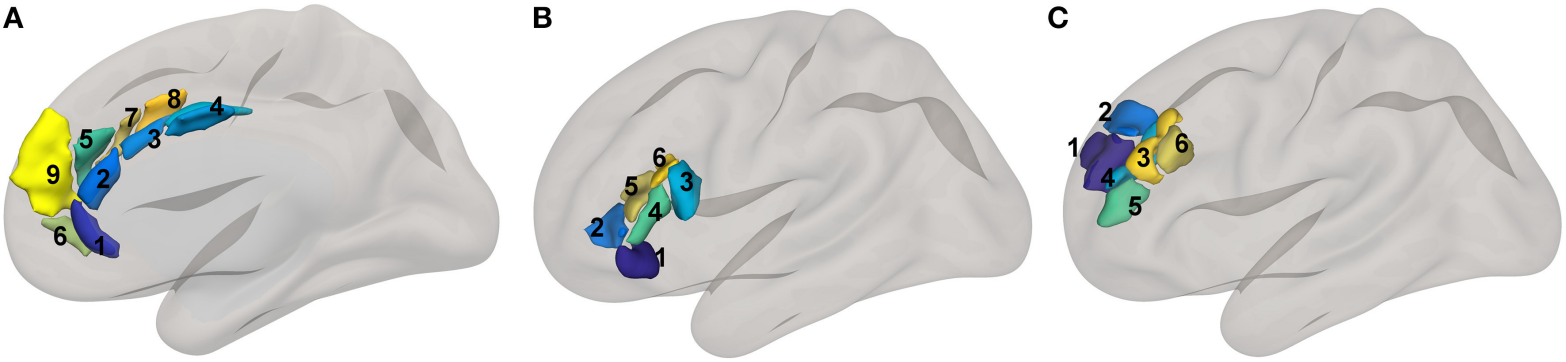
Cognitive control network ROI locations. ROIs based upon a subset of regions implicated in control in the high-dimensional atlas Human Connectome Project Multimodal Atlas (13). Surface-space ROIs were converted into volumetric Montreal Neurologic Institute Atlas space with FreeSurfer (14). The prefix “a” or “p” typically denotes an anterior or posterior subregion, but sometimes this adjective is valid only within a smaller parcellation of a larger unimodally-defined subregion. The same is true for “d,” “v, “r,” “m,” “l,” which typically stand for “dorsal,” “ventral,” “rostral,” “medial,” “and “lateral,” respectively. **(A)** midline cognitive control regions. Cingulate subregions: 1. a24, 2. p24, 3. a24pr, 4. p24pr; Paracingulate subregions: 5. d32, 6. p32, 7. a32pr, 8. p32pr, 9. 9m. **(B)** ventrolateral cognitive control regions: 1. 47l, 2. p47r, 3. 44, 4. 45, 5. IFSa, 6. IFSp. **(C)** dorsolateral cognitive control regions: 1. 9a, 2. 9p, 3. 9-46d, 4. a9-46v, 5. p9-46v, 6. 46.

ROIs used in this study came from several sources. Cortical reward network (Figure 1) and cognitive control network (Figure 2) ROIs were based on the Human Connectome Project Multimodal Atlas (13). The basal ganglia ROIs were based on the frontoparietal control network striatal subregion from a functional connectivity-based striatal atlas (7-network parcellation) (33). Thalamic ROIs were based on the Anatomy Toolbox (34); we used the mask of the thalamic subregion that projects to PFC, thresholded at 50% probability. ROIs also included the anterior and posterior hippocampus regions implicated in PTSD by Chen and Etkin (35). Centromedial and basolateral sections of the amygdala [SPM Anatomy Toolbox (34), thresholded at 50%] were selected because of their role in fear learning and threat detection (36, 37). Human Connectome Project surface-space ROI definitions were propagated to volumetric MNI152 atlas space via Freesurfer (14) to permit inclusion of subcortical and cortical regions in ROI-to-ROI functional connectivity models in CONN. See Table 2 for a complete list of ROIs and Figures 1, 2 for ROI visualizations.

**Table 2:** Regions of Interest (ROIs).

**Network**	**Anatomical group**	**ROI**
**SUBCORTICAL**		
	MTL	Amygdala (CM)^a^
		Amygdala (BL)^a^
		Ant. Hippocampus^b^
		Pos. Hippocampus^b^
	Basal Ganglia and Thalamus	Striatum (FPN)^c^
		Thalamus (PFC)^a^
**REWARD**^**d**^		
	MPFC	10r, 10v, 10pp
	Orbital and Polar	47s, 47m, a47r
		11l, 13l
		a10p, p10p
		OFC, Pofc
	Subgenual	25, s32
**COGNITIVE CONTROL**^**d**^		
	Ant. Cingulate	a24, p24
		a24pr, p24pr
	Ant. Paracingulate	d32, p32
		a32pr, p32pr
	Dorsomedial PFC	9m
	Inf. Frontal Cortex	47l, p47r
		44, 45
		IFSa, IFSp
	Dorsolateral PFC	9a, 9p
		9-46d, a9-46v, p9-46v
		46

*Note that ROIs based upon the Human Connectome Project Multimodal Atlas are a subset of a high-dimensional atlas. As a result, while the prefix “a” or “p” typically denotes an anterior or posterior subregion, sometimes this adjective is valid only within a smaller parcellation of a larger unimodally-defined subregion. The same is true for “d”,”v”,”r”,”m”,”l”, which typically stand for ”dorsal”,”ventral”, “rostral”,”medial”,”and “lateral”, respectively. Please see Figures 1, 2 for visualizations of cortical ROIs and the original references for precise ROI descriptions. The abbreviations CM and BL in the subcortical ROIs refer to the centromedial and basolateral divisions of the amygdala, respectively*.

a *For ROI details see (34)*.

b *For ROI details see (35)*.

c *For ROI details see (33)*.

d *For ROI details see (13)*.

### ROI-to-ROI Functional Connectivity Analyses

All statistical analyses were conducted with either MATLAB (v.17b, Mathworks, Natick MA) or the CONN Toolbox. Residuals from preprocessing were entered into subject-level models of ROI-to-ROI connectivity. Prior to second-level modeling, a hierarchical multinomial logistic regression was first run to determine whether the continuous variables of age, sex, depression severity, or PTSD symptom severity influenced suicide scores (IDS-SR Item #18) and thus should be included as covariates in subsequent models for hypothesis testing. Examination of model coefficient *p*-values indicated that these potential covariates were not predictive of suicidality scores (all *p* > 0.1), however, a binomial logistic regression to determine whether these variables influenced the risk of having any suicidal thoughts and behaviors (operationalized categorically as IDS-SR Item #18>0) revealed that both age (*p* = 0.04) and depression severity (*p* = 0.02) were significant predictors. Thus, we included age and depression severity as covariates in all ROI-to-ROI functional connectivity analyses, along with a covariate for scanner model (Siemens TimTrio or Prisma).

Second-level ROI-to-ROI functional connectivity models were constructed using the CONN Toolbox. The simple main effect of the IDS-SR suicide item (#18) from a model including subject, age, scanner, IDS-SR #18, and total IDS-SR score as predictor variables was used to identify ROI pairs where connectivity was influenced by suicidality at the seed-level false discovery rate-corrected (38) threshold of *p*-FDR < 0.05. Functional connectivity betas from significant ROI pairs were then inspected for heteroscedasticity. Residuals were produced by orthogonalizing betas to model factors and were then plotted against Item #18 scores to identify potential outliers. Quality control inspections did not identify univariate or bivariate outliers. Though initial testing did not find that PTSD symptoms influenced suicidality, we confirmed this by computing correlations between subject-level coefficients describing ROI-to-ROI connectivity and PCL total scores. Finally, follow-up two-sample *t*-tests were used to examine group differences in connectivity (patients with IDS-SR Item 18 >1, patients with IDS-SR Item 18 = 1, non-suicidal patient controls, non-suicidal healthy controls) between ROI pairs tracking suicidality. These *post-hoc* tests were considered significant at the Bonferroni-corrected threshold of *p* < 0.008.

Importantly, while our metric of suicidality reflects self-reported suicidal thoughts at the time of imaging, the results of our neuroimaging analyses may also reflect more stable trait- rather than state-based correlates of suicidality. Thus, we used similar methods to evaluate differences in functional connectivity by lifetime history of suicidality per review of participants' clinical charts. Second-level models were constructed to evaluate ROI-to-ROI connectivity comparing those with (*n* = 30) and without (*n* = 20) prior psychiatric hospitalization for suicidal risk. Age, scanner (3T Siemens TimTrio or 3T Siemens Prisma), current PTSD severity, and current depression severity were included in models as covariates.

### Morphometry Analyses

Morphometry analyses were conducted using FreeSurfer v.5.3.0. (14). During cortical reconstruction, each subject's surface-space cortical thickness map was registered to FreeSurfer fsaverage space, a template space that approximates the MNI152 atlas (14). Average cortical thickness in millimeters was extracted from ROIs in which there was a significant relationship between functional connectivity and suicidality. GLMs including cortical thickness, age, sex, depression, and scanner type were included as potential predictors of ROI-to-ROI functional connectivity in statistical models examining structure-function relationships. If statistically significant effects of thickness were observed, betas corresponding to structural measures were plotted against functional connectivity values to identify potential bivariate or univariate outliers (normalized betas> ±2.5 SD). All bivariate outliers were removed from the analysis. If univariate outliers were identified, results are reported with and without outlier observations.

## Results

### Reward Processing Networks

**Figure 3:**
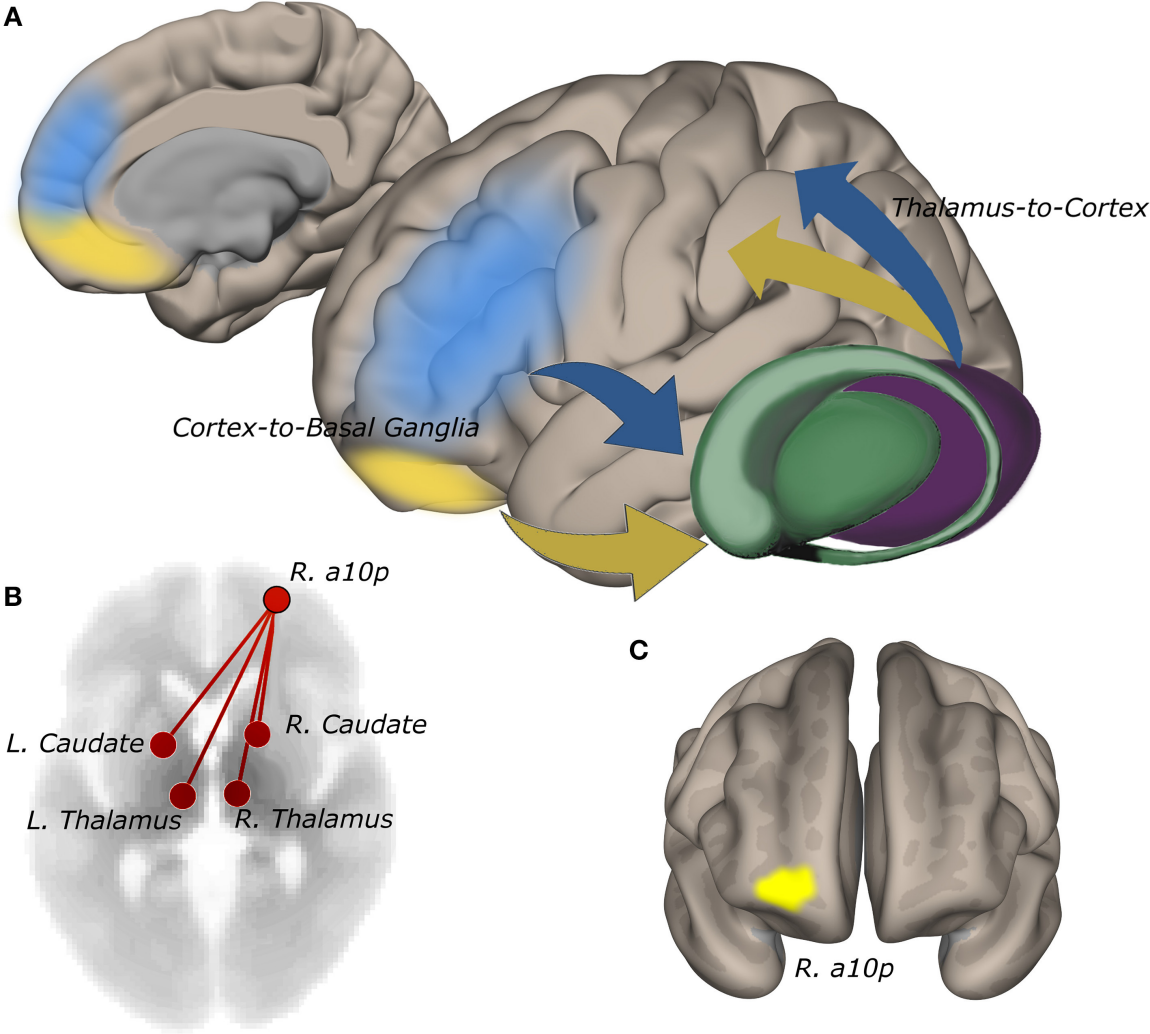
Functional connectivity of the right anterior frontopolar cortex to the striatum and thalamus tracks self-reported suicidality. **(A)** cortico-basal ganglia-thalamic circuits involved in reward and cognitive control. Information from reward (yellow) and cognitive control (blue) networks guide decision-making. Network signals converge anatomically in the striatum via cortico-basal ganglia-thalamic projections (19, 20). Abnormalities in cognitive and reward circuits may impair integrative processing of goal and reward information giving rise to maladaptive decision-making in suicidality. **(B)** connectivity of right a10p in the anterior frontal pole to bilateral thalamus and striatum is positively correlated with IDS-SR #18 rating (*p*-FDR = 0.05). **(C)** the a10p ROI in cytoarchitecturally-defined lateral frontopolar area 1 (39) is recruited by simulated gambling tasks (13).

**Figure 4:**
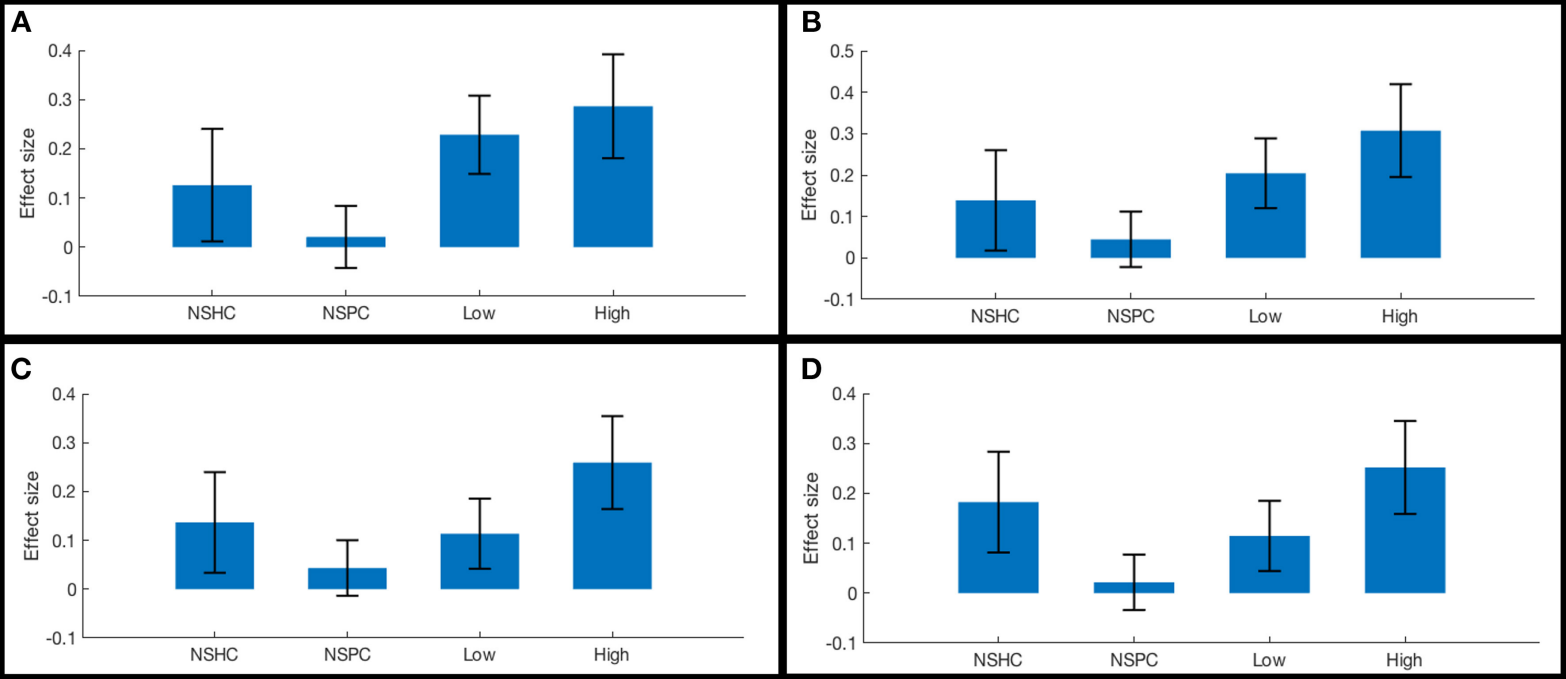
Functional connectivity in reward network ROI pairs by self-reported current suicidality. Bars illustrate mean effect sizes and 90% confidence intervals associated with the right anterior frontal pole seed by group. NSHC, non-suicidal healthy controls; NSPC, non-suicidal patient controls (PTSD diagnosis with IDS-SR Item #18 = 0); Low = PTSD with IDS-SR Item #18 = 1, and High = PTSD with IDS-SR Item #18 > 1. Between-group differences are not significant after multiple comparisons correction. **(A)** right anterior frontal pole connectivity to left caudate. **(B)** right anterior frontal pole connectivity to right caudate. **(C)** right anterior frontal pole connectivity to left thalamus. **(D)** right anterior frontal pole connectivity to right thalamus.

ROI-to-ROI functional connectivity of region “a10p” (see Table 2), in the right anterior frontal pole to the caudate and thalamus in each hemisphere tracked suicidality (Figure 3). A simple main effect of suicidality was observed in right a10p connectivity to the right centromedial amygdala [*t*_(60)_ = 4.03, *p* < 0.05], dorsomedial thalamus (right [*t*_(60)_ = 3.35, *p* = 0.05]; left [*t*_(60)_ = 3.21, *p* = 0.05]), and striatum [right: *t*_(60)_ = 3.32, *p* = 0.05; left: *t*_(60)_ = 3.14, *p* = 0.05]. *Post hoc* testing indicated that differences in amygdala connectivity were driven by PTSD symptoms [t_(63)_ = 2.11, *p* < 0.05], thus we did not examine this ROI further. Functional connectivity of right a10p to the remaining ROIs was not influenced by PTSD symptoms (all *p* > 0.1). Though our exploratory *post-hoc* testing did not find evidence of statistically reliable differences between groups after Bonferroni correction, connectivity between right a10p and the striatal and thalamic ROIs was particularly low in non-suicidal individuals with PTSD (all uncorrected *p* < 0.05). See Figures 4A–D for plots of subgroup comparisons.

Initial tests indicated that right a10p thickness was negatively associated with suicidality effects on functional connectivity to both the left (*t* = −3.35, *p* = 0.001) and right striatum (*t* = −3.13, p = 0.002). However, these results were not significant after the removal of one bivariate outlier (left striatum: *t* = −1.68, *p* = 0.1; right striatum: *t* = −1.85, *p* = 0.07).

When examining effects of previous history, we observed no significant differences between those with and without prior hospitalizations for suicidality in the reward network (all *p* > 0.1).

### Cognitive Control Networks

**Figure 5:**
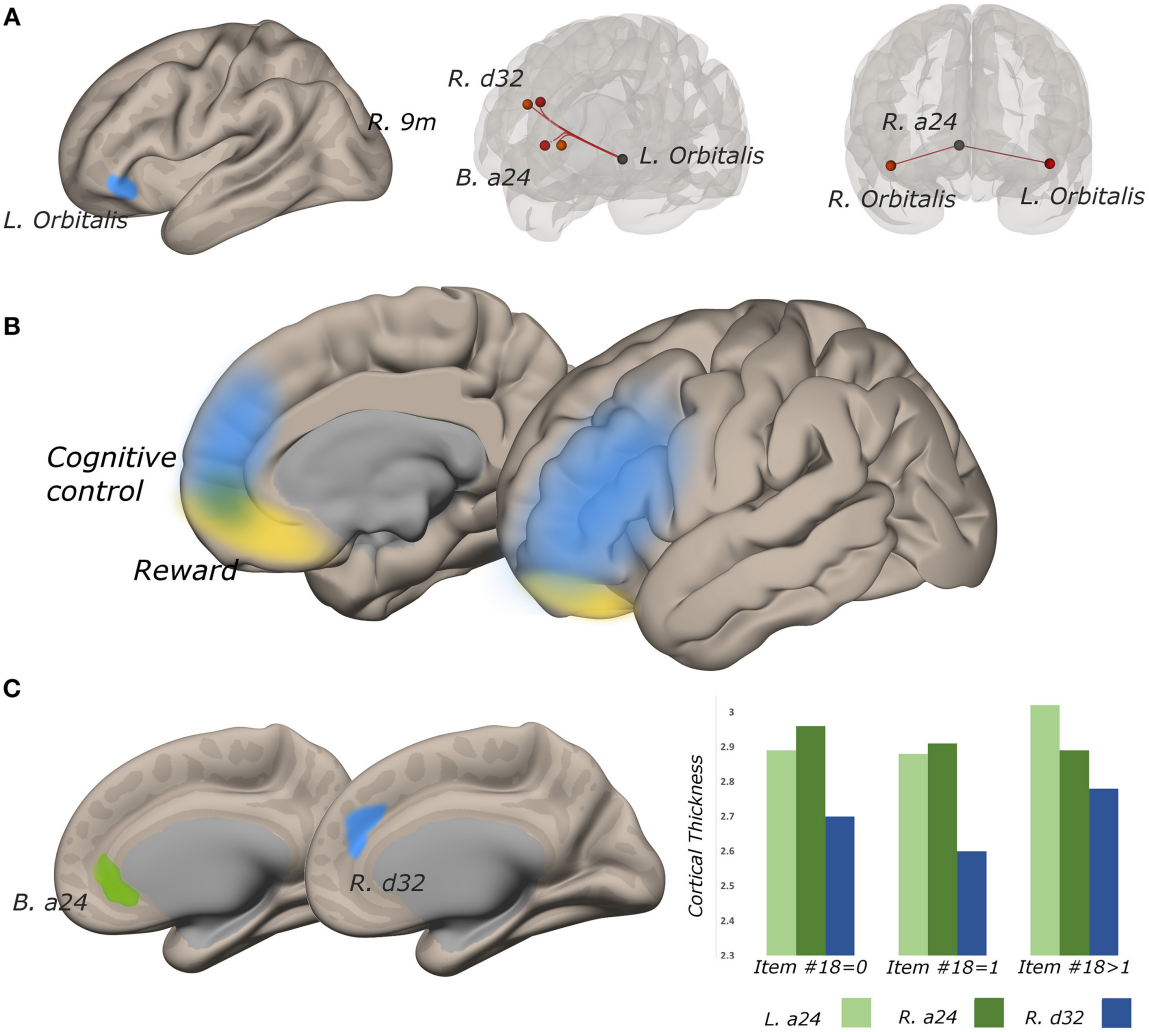
Functional connectivity strength between lateral pars orbitalis and midline cognitive control regions tracks suicidality, and cortical thickness is predictive of significant connectivity relationships. **(A)** left lateral pars orbitalis ROI (left) was more strongly connected to ROIs in bilateral a24, right d32, and right 9m (all *p*-FDR < 0.05), in those with more severe suicidality (middle). Connectivity strength of right a24 to bilateral orbitalis also tracks suicidality (*p*-FDR < 0.05) (right). **(B)** reward and cognitive control networks overlap in perigenual cortex. **(C)** cortical thickness in a24 predicts the relationship between suicidality and orbitalis-to-a24 connectivity in the ipsilateral hemisphere. Right d32 thickness predicted the relationship between a24-to-left lateral orbitalis connectivity and suicidality.

**Figure 6:**
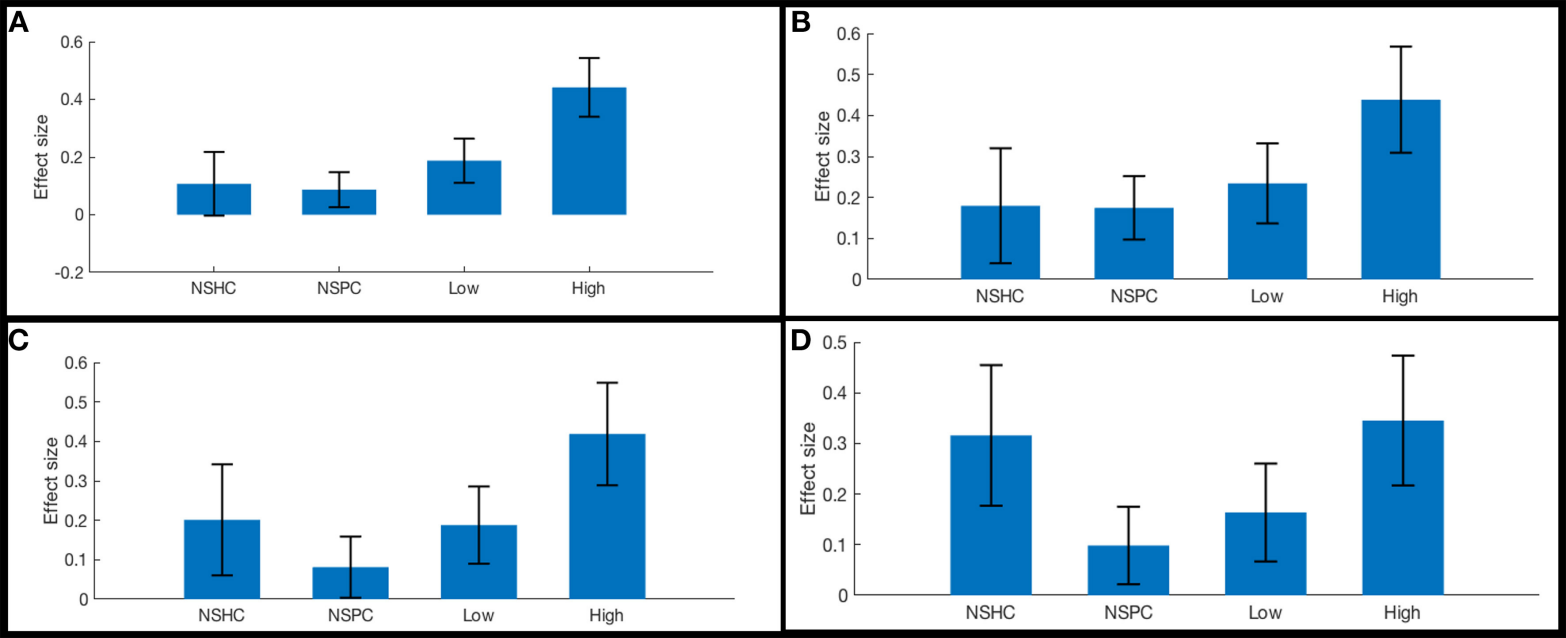
Functional connectivity in cognitive control network ROI pairs by self-reported current suicidality. Bars illustrate mean effect sizes and 90% confidence intervals associated with lateral orbitals seeds by group. NSHC, non-suicidal healthy controls; NSPC, non-suicidal patient controls (PTSD diagnosis with IDS-SR Item #18 = 0), Low = PTSD with IDS-SR Item #18 = 1, and High = PTSD with IDS-SR Item #18 > 1. **(A)** left orbitalis-to-right dorsogenual BA 32. Connectivity differences between high- and low-suicidality, and high-suicidality vs. non-suicidal patients are significant after Bonferroni correction [both *t*_(58)_ >5.0, *p* < 0.008]. **(B)** left orbitalis-to-left anterior BA 24. **(C)** left orbitalis-to-right anterior BA 24. **(D)** right orbitalis-to-right anterior BA 24.

The functional connectivity of the lateral subregion of pars orbitalis to midline PFC regions involved in monitoring the demand for cognitive control, was influenced by suicidality (Figure 5). Left lateral pars orbitalis was more strongly connected to the a24 [left: *t*_(60)_ = 3.28, *p*-FDR < 0.05; right: *t*_(60)_ = 4.32, *p* < 0.005], d32 [*t*_(60)_ = 5.58, *p* < 0.0001), and right 9m ROIs [t_(60)_ = 3.38, *p* < 0.05], in the rostral portion of the anterior cingulate gyrus, dorsal paracingulate, and medial section of Brodmann's Area 9, respectively. Our *post hoc* tests indicated that connectivity relationships were not influenced by PTSD severity. Follow-up tests indicated that group differences in left orbitalis-to-d32 connectivity between high- and low-suicidality, and high-suicidality vs. non-suicidal patients were significant after Bonferroni correction [both *t*_(58)_ > 5.0, *p* < 0.008], with functional connectivity being strongest in the high suicidality patients in each comparison. All other follow-up comparisons were non-significant. See Figures 6A–D for plots of connectivity means.

Functional connectivity between left lateral orbitalis and left a24 was positively associated with cortical thickness in the cingulate ROI [*t*_(60)_ = 2.31, *p* < 0.05]. Left orbitalis-to-right d32 connectivity was associated with right d32 thickness (*t* = 3.10, *p* < 0.005). *Post hoc* examination of the residuals from both thickness analyses identified univariate outliers, but effects remained significant even after outlier removal (all *p* < 0.05).

In the right hemisphere, functional connectivity of a24 to right lateral pars orbitalis tracked suicidality [*t*_(60)_ = 3.52, *p* < 0.05]. This ROI-to-ROI relationship was also positively associated with cortical thickness in right a24 [t_(60)_ = 2.04, *p* < 0.05)] Heteroscedasticity assessment revealed one univariate outlier, however, this structure-function relationship remained significant even after outlier removal [*t*_(60)_ = 2.25, *p* < 0.05].

Similar to our results in reward networks, ROI-to-ROI functional connectivity in cognitive control network regions did not track past psychiatric hospitalization.

## Discussion

To our knowledge, this is the first study to evaluate the relationship between suicidality and the structural and functional integrity of decision-making circuits, in individuals with PTSD. We found that functional connectivity relationships in the reward and cognitive control networks, two functional networks involved in decision-making, tracked self-reported suicidality. Additionally, in several cases, cortical thickness in subregions of the cingulate and paracingulate cortices predicted the relationship between suicidality and ROI-to-ROI functional connectivity in cognitive control regions. These findings provide preliminary support for the previously extended hypothesis that suicidality emerges from parallel dysfunction in ventral PFC reward and PFC cognitive control networks (40), and extends the association between decision-making and suicide in depression to PTSD (16, 26).

### Ventral PFC Reward Network

In our study, suicidality was positively correlated with functional connectivity between right a10p in the anterior frontopolar cortex, to the striatum and thalamus. This finding is consistent with previous reports of elevated resting cerebral glucose metabolism in the ventral PFC and striatum of prior suicide attempters with depression (17).

The alignment of our functional connectivity results with previous task-based neuroimaging findings is more complex. Typically, network functional connectivity strength is predictive of univariate fMRI activation on task (41, 42). In line with this heuristic, suicidality and functional connectivity between a10p and the striatum and thalamus were positively correlated in this study, complementing previous reports of stronger OFC fMRI activation for gambling wins over losses in past attempters (12). However, the positive correlation observed here is at odds with task-based fMRI reports of hypoactivity in the reward circuit during probabilistic decision-making in suicidality and depression (11). This discrepancy may be driven strictly by differences in methodology or psychological state (resting vs. on task). Alternatively, strong, tonic resting connectivity in the reward circuit may reduce specificity of event-related firing. This loss of specificity may give rise to hypoactivation in a univariate contrast.

### Cognitive Control Networks

In this study, suicidal severity and functional connectivity between the lateral pars orbitalis and midline cognitive control regions was positively correlated. Additionally, our morphometry results indicated that thickness in subregions of cingulate and paracingulate cortex influenced these connectivity relationships.

The pars orbitalis has been implicated in elaborative memory processing at both encoding and retrieval (43), while midline PFC regions contribute to broad range of control functions involved in value appraisal, monitoring, and regulatory control (44, 45). In patients contemplating suicide, strong functional connectivity between these areas may promote the development of enduring, but potentially biased, memories that influence decision-making in ways that foster maladaptive behavior. For instance, if greater salience is assigned to positive or negative reinforcements during value appraisal, strong functional connectivity between cingulate/paracingulate and orbitalis may promote the encoding of biased memory representations that misrepresent decision-making consequences. In line with this hypothetical, fMRI studies employing gambling paradigms have found evidence of hyper-responsivity to wins in rostral ACC (12) but insensitivity to relative risk in pars orbitalis (8), in depressed individuals with history of suicide attempts. We speculate that strong functional connectivity between these areas may facilitate engagement in high-risk behaviors or perhaps lead individuals to misjudge the severity of their own developing suicidality.

Similarly, strong functional connectivity between orbitalis and midline control regions may facilitate negative emotional biases and the development of the feelings of alienation and perceived burdensomeness that accompany suicidality (46). Several studies have found evidence of a bias in left orbitalis fMRI activation toward the processing of negative affective stimuli in previous suicide attempters (12, 47). Similar biases have been observed in dorsal ACC in adolescent suicide attempters with depression (48).

The follow-up between-group contrasts of cognitive control network connectivity yield insights relevant to the important, emerging topic of distinct suicidal biotypes (49–51). Notably, functional connectivity between orbitalis and anterior cingulate/paracingulate was strongest in patients self-reporting greater suicidality. Results from studies contrasting decision-making between ideators vs. attempters, or those with histories of high- vs. low-lethality attempts, have found evidence linking subgroups to unique biosignatures and patterns of decision-making (26). In several studies of depressed older adults, delay discounting was exaggerated in those with previous low-lethality attempts, but those with previous high-lethality attempts displayed above-average ability to delay gratification (9, 10). In the case suicidality, better cognitive control may enable individuals to plan attempts more likely to result in completion, and may serve as a biomarker of a high-risk suicidal subtype. This view implies that in some cases, poor cognitive control may paradoxically be protective, at least against high-lethality attempts. Importantly, control network connectivity was consistently lowest in patients with PTSD without self-reported suicidality in this study. Thus, the findings of this study complement the emerging dialogue surrounding multiple phenotype-diathesis models of suicidality (50, 51), and underscore the need for empirical testing of these models.

## Limitations

This study has several limitations common to secondary, cross-sectional data analyses. First, data used in these analyses were from studies that were not specifically designed to address suicidality, thus only one suicidality measure was obtained. The number of patients in severity-based subgroups was also unbalanced.

Co-morbid depression may potentially exert residual influence on our results despite statistical covariance for depression. Our sample of patients was selected based on clinical diagnosis of PTSD however depression was highly comorbid in our sample.

We also note that our power to interpret the relationship of our functional connectivity results to the broader decision-making and suicidality literature is limited by the use of resting state data, and the lack of a behavioral measure of decision-making. This precludes direct comparison of our results to prior work in suicidality in depression. Despite this limitation, the correlates of suicidality identified in this study are consistent with previous observations.

Finally, we note that while promising, like many studies conducted in clinical populations, statistical power is an issue. In the current preliminary study, multiple comparisons correction was applied at the seed-level to control for type I error without undue inflation of type II error in a small sample study. These preliminary results await replication in a larger population with the application of more stringent analysis-wise correction procedures.

## Conclusions

We observed neural correlates of suicidality in two networks involved in decision-making, the reward and cognitive control networks, in a naturalistic sample of patients with PTSD. These results complement prior imaging findings related to suicide in depressed individuals, underscoring the potential of decision-making correlates as transdiagnostic biomarkers of suicidality. This advance is important given the urgent need for objective markers of risk, in light of the current suicide public health crisis. Further investigations are needed to evaluate whether these results will extend to a study designed to specifically examine suicidality in PTSD, and to other high-risk disorders and conditions e.g., (52, 53). Future work should also investigate whether these biomarkers can be used to prospectively identify individuals at-risk for suicide.

## Author Contributions

JB conceived of this secondary study and carried out the analyses. LLC and NSP conducted the original studies supplying data used for these analyses. JB, EA, and NSP wrote the manuscript, to which the other authors made important intellectual contributions. All authors approved of the submitted manuscript.

### Conflict of Interest Statement

NSP and LLC have received grant support from Neuronetics, Neosync and Cervel Neurotech, and LLC has been a consultant for Magstim. The remaining authors declare that the research was conducted in the absence of any commercial or financial relationships that could be construed as a potential conflict of interest.
