# Supplementary material for: Neuroimaging Correlates of Suicidality in Decision-Making Circuits in Posttraumatic Stress Disorder
Source: Front Psychiatry. 2019 Feb 12;10:44. doi: 10.3389/fpsyt.2019.00044 (PMC6379274; doi:10.3389/fpsyt.2019.00044)
Supplement: Supplementary file 1 [file Table_1.docx]

**Supplementary Data for Neuroimaging Correlates of Suicidality in Decision-Making Circuits in Posttraumatic Stress Disorder**

Jennifer Barredo, PhD^1,2^, Emily Aiken, MA^1^, Mascha van ’t Wout-Frank, PhD^1,2^, Benjamin D. Greenberg, MD PhD^1,2,3^, Linda L. Carpenter, MD^1,2,3^, Noah S. Philip, MD^1,2,3^

^1^ Center for Neurorestoration and Neurotechnology, Providence VA Medical Center, Providence, RI

^2^ Dept. of Psychiatry and Human Behavior, Alpert Medical School of Brown University, Providence, RI

^3^ Butler Hospital Neuromodulation Research Facility, Providence, RI

**SUPPLEMENTARY INFORMATION**

**Table S1. Concurrent Psychopharmacology in Patients with PTSD.** The “High” suicidality group includes patients with IDS-SR item #18 scores greater than one. The “Low” group corresponds to patients with an item score equal to one, and the “No” group is comprised of patients with scores equal to zero. Full medication data were unavailable for six subjects with PTSD.

| **Group** | **Current Medication (total daily dose in mg)** |
| --- | --- |
| High | Armodafinil 250 mg |
|  | Bupropion 120 mg |
|  | Duloxetine 120 mg |
|  | Mirtazapine 15 mg |
| High | Clonazepam 3 mg |
| High | Bupropion 200 mg |
|  | Citalopram 40 mg |
|  | Risperidone 1.5 mg |
|  | Trazodone 200 mg |
| High | Trazodone 200 mg |
|  | Venlafaxine 100 mg |
| High | Mirtazapine 45 mg |
|  | Nortriptyline 100 mg |
|  | Trazodone 150 mg |
|  | Venlafaxine 450 mg |
| High | None |
| High | Aripiprazole 10 mg |
|  | Bupropion 450 mg |
|  | Melatonin 3 mg |
|  | Prazosin 3 mg |
|  | Trazodone 100 mg |
|  | Venlafaxine 300 mg |
| High | Clonazepam 2.5 mg |
| Low | Bupropion 300 mg |
|  | Diazepam 20 mg |
|  | Duloxetine 30 mg |
|  | Gabapentin 3600 mg |
|  | Prazosin 15 mg |
|  | Ziprasidone 20 mg |
| Low | Bupropion 400 mg |
|  | Dextroamphetamine 60 mg |
|  | Gabapentin 2400 mg |
|  | Lorazepam 1.5 mg |
|  | Mirtazapine 45 mg |
|  | Trazodone 150 mg |
| Low | Clonazepam 4 mg |
| Low | Zolpidem 10 mg |
| Low | Gabapentin 3600 mg |
|  | Lithium 900 mg |
|  | Lorazepam 0.5 mg |
|  | Quetiapine 400 mg |
| Low | Zolpidem 10 mg |
| Low | Fluoxetine 20 mg |
|  | Trazodone 25 mg |
| Low | Amphetamine/Dextroamphetamine 60 mg |
|  | Bupropion 300 mg |
|  | Duloxetine 90 mg |
| Low | BupreNorphine 16 mg/Naloxone 4 mg |
|  | Clonazepam 0.5 mg |
|  | Melatonin 5 mg |
|  | Venlafaxine 300 mg |
| Low | None |
| Low | Alprazolam 3 mg |
|  | Clonidine 0.2 mg |
|  | Desvenlafaxine 100 mg |
|  | Dextroamphetamine 80 mg |
| Low | None |
| Low | Bupropion 300 mg |
|  | Lamotrigine 200 mg |
|  | Methylphenidate 15 mg |
|  | Risperidone 2 mg |
|  | Trazodone 25 mg |
|  | Venlafaxine 300 mg |
| Low | Dexmethylphenidate 10 mg |
|  | Duloxetine 30 mg |
|  | Lorazepam 2 mg |
|  | Vortioxetine 20 mg |
| Low | Amphetamine salts 25 mg |
|  | Pregabalin 150 mg |
|  | Quetiapine 25 mg |
|  | Vortioxetine 30 mg |
| No | Citalopram 20 mg |
|  | Trazodone 100 mg |
| No | Gabapentin 1800 mg |
|  | Hydroxyzine 25 mg |
|  | Quetiapine 200 mg |
|  | Tranylcypromine 60 mg |
| No | Aripiprazole 5 mg |
|  | Sertraline 200 mg |
| No | Sertraline 50 mg |
|  | Trazodone 100 mg |
| No | Fluoxetine 40 mg |
|  | Lamotrigine 150 mg |
|  | Naltrexone 50 mg |
|  | Prazosin 2 mg |
|  | Quetiapine 300 mg |
| No | Quetiapine 125 mg |
| No | Sertraline 200 mg |
|  | Trazodone 100 mg |
|  | Valproic Acid 1500 mg |
| No | None |
| No | Bupropion 300 mg |
|  | Dextroamphetamine 15 mg |
|  | Lorazepam 0.5 mg |
| No | Alprazolam 3 mg |
|  | Lisdexamfetamine 40 mg |
|  | Venlafaxine 150 mg |
| No |  |
| No | Armodafinil 250 mg |
|  | Bupropion 450 mg |
|  | Duloxetine 120 mg |
|  | Mirtazapine 15 mg |
| No | Amphetamine salts 10 mg |
|  | Bupropion 150 mg |
|  | Clonazepam 1 mg |
|  | Trazodone 50 mg |
|  | Ziprasidone 60 mg |
| No | Gabapentin 600 mg |
|  | Methadone 135 mg |
|  | Venlafaxine 150 mg |
| No | Buspirone 5 mg |
|  | Citalopram 15 mg |
|  | Lorazepam 2 mg |
|  | PropraNolol 5 mg |
| No | Prazosin 5 mg |
|  | Naltrexone 380 mg/inj |
|  | Sertraline 100 mg |
| No | Sertraline 50 mg |
| No | None |
| No | None |
| No | Trazodone 50 mg |
|  | Venlafaxine 300 mg |
| No | Topiramate 100 mg |
| No | Quetiapine 100 mg |
|  | Trazodone 50 mg |
